# Transforming growth factor‐beta renders ageing microglia inhibitory to oligodendrocyte generation by CNS progenitors

**DOI:** 10.1002/glia.23612

**Published:** 2019-03-12

**Authors:** Roey Baror, Björn Neumann, Michael Segel, Kevin J. Chalut, Stephen P. J. Fancy, Dorothy P. Schafer, Robin J. M. Franklin

**Affiliations:** ^1^ Wellcome‐MRC Stem Cell Institute University of Cambridge Cambridge United Kingdom; ^2^ Department of Paediatrics University California San Francisco San Francisco California; ^3^ Department of Neurobiology and the Brudnik Neuropsychiatric Institute University of Massachusetts Medical School Worcester Massachusetts

**Keywords:** ageing, extracellular matrix, microglia, oligodendrocyte, progenitor cells

## Abstract

It is now well‐established that the macrophage and microglial response to CNS demyelination influences remyelination by removing myelin debris and secreting a variety of signaling molecules that influence the behaviour of oligodendrocyte progenitor cells (OPCs). Previous studies have shown that changes in microglia contribute to the age‐related decline in the efficiency of remyelination. In this study, we show that microglia increase their expression of the proteoglycan NG2 with age, and that this is associated with an altered micro‐niche generated by aged, but not young, microglia that can divert the differentiation OPCs from oligodendrocytes into astrocytes in vitro. We further show that these changes in ageing microglia are generated by exposure to high levels of TGFβ. Thus, our findings suggest that the rising levels of circulating TGFβ known to occur with ageing contribute to the age‐related decline in remyelination by impairing the ability of microglia to promote oligodendrocyte differentiation from OPCs, and therefore could be a potential therapeutic target to promote remyelination.

## 1 INTRODUCTION

Remyelination is an important regenerative process in the CNS in which new myelin sheaths are restored to demyelinated axons by oligodendrocytes which are newly generated by adult oligodendrocyte progenitor cells (OPCs) (Franklin and ffrench‐Constant, 2017). This results in the restoration of saltatory conduction, which is lost during demyelination, and protection from irreversible axonal atrophy (Irvine & Blakemore, 2008; Smith, Blakemore, & Mcdonald, 1979). Remyelination is controlled by a complex interplay of multiple molecules and pathways that have either facilitatory or inhibitory effects on the proliferation, migration and differentiation of adult OPCs (Franklin and ffrench‐Constant, 2017). One of the main rate limiting steps in remyelination is the efficiency of OPC differentiation. This is especially evident in multiple sclerosis (MS), a chronic demyelinating disease often of several decades' duration: chronic demyelinated lesions frequently contain oligodendrocyte lineage cells that have failed to undergo full differentiation into myelin sheath‐forming cells (Chang, Tourtellotte, Rudick, & Trapp, 2002; Kuhlmann et al., 2008; Wolswijk, 1998).

The molecular mediators of remyelination are produced by a variety of cell types present within demyelinating lesions. These include microglia, the resident immune cells of the CNS, which are now recognized to play a major role in CNS remyelination (Lloyd & Miron, 2016; Rawji, Mishra, & Yong, 2016). Microglia affect OPC differentiation rate through the clearance of myelin debris (which is inhibitory to OPC differentiation) (Kotter, Li, Zhao, & Franklin, 2006; Natrajan et al., 2015) and by secreting pro‐regenerative factors (Miron & Franklin, 2014; Miron et al., 2013).

Previous studies have identified the extracellular matrix (ECM) as a critical factor regulating OPC differentiation during remyelination (Lau et al., 2012). For example, chondroitin sulfate proteoglycans (CSPGs), which are deposited by different cell types in demyelination lesions, are inhibitory to OPC differentiation and remyelination (Keough et al., 2016). Since microglia cells compose a large component of the cells present within demyelinating lesions, it is conceivable that they too contribute to the ECM composition of a lesion. To test this, we have developed a protocol for capturing the ECM environment associated with microglia (the micro‐niche). Moreover, since TGFβ levels increase with ageing and have been shown to negatively affect hippocampal neurogenesis in the CNS (Buckwalter et al., 2006; Carlson et al., 2009; Doyle, Cekanaviciute, Mamer, & Buckwalter, 2010), we hypothesized that TGFβ‐mediated effects on the microglial micro‐niche might contribute to the age‐related decline in the efficiency of remyelination (Cantuti‐Castelvetri et al., 2018; Miron et al., 2013; Ruckh et al., 2012; Shields, Gilson, Blakemore, & Franklin, 1999; Sim, Zhao, Penderis, & Franklin, 2002).

## 2 MATERIALS AND METHODS

### 2.1 Animal husbandry

Only Sprague Dawley rats were used for this study. Rats were bred and raised at the Animal Facility at the University of Cambridge. All animals were fed standard diet and were kept under 12 hr cycles of light and darkness. All animals used for experiments were sacrificed by schedule 1 approved methods, according to the requirements and regulations set by the United Kingdom Home Office.

### 2.2 Induction of white matter demyelination

In order to induce demyelination, female Sprague–Dawley rats (Harlan Laboratories) of 18 months of age were used. The rats were anesthetized with buprenorphine (0.03 mg/kg, s.c.) and 2.5% isoflurane. Demyelination was induced by stereotaxic injection of 4 μL of 0.01% ethidium bromide (EB) into the caudal cerebellar peduncles (CCPs), as previously described (Woodruff & Franklin, 1999). EB was delivered at a rate of 1 μL/min. After EB delivery the injection needle remained in position for additional 4 min.

### 2.3 Isolation and culture of aged and young microglia

Neonatal (P0‐P20), young adult (P30‐P90), and aged (18–24 months) rats were decapitated after lethal injection of phenobarbital. Brains were removed shortly after death (including cerebrum and cerebellum) and placed into ice cold HALF isolation medium (Hibernate A Low Fluorescence), supplemented with 2% of B27. Meninges, olfactory bulbs and remains of spinal cord (if present) were removed and brain tissue was chopped mechanically into 1 mm pieces using sterile scalps. The minced tissue was spun down in 100 g for 2 min in RT. Brain tissue was then suspended in dissociation solution (34 U/mL papain [Worthington], 20 μg/mL DNAse Type IV [Gibco] in HALF) for 30 (neonatal) to 40 (adult) min in 37°C, on a shaker set to 55 RPM. Digestion was stopped by adding cold HBSS (Hank's Balanced Salt Solution, no magnesium/calcium; Gibco). The tissue was centrifuged for 5 min, in 250 g. Supernatant was completely aspirated and tissue was re‐suspended in neutralizing solution (HALF+ 2% B27+ 2 mM sodium‐pyruvate) for 5 min in RT. In order to create a single cell suspension, cells were first titurated using a 5 mL serological pipette and subsequently three fire polished glass pipettes in decreasing opening sizes. After each trituration step the tissue suspension was allowed to sediment and the supernatant, containing the cells, was transferred into a fresh tube, while being passed through a 70 μm cell strainers to ensure single cell suspension. After removing the supernatant each time, 2 mL of fresh neutralizing solution were added to the remaining tissue. After the whole tissue was passed though the cell strainer, a 90% pre‐filtered (20 μn filters) isotonic Percoll solution (GE Healthcare, diluted in 10× PBS pH 7.2 [Gibco]) was added. Final Percoll concentration of 22.5% was achieved by adding phenol‐red free DMEM/F12 (Dulbecco's Modified Eagle Medium/Nutrient Mixture F‐12; Gibco). The single cell suspension was then centrifuged for 20 min in 800 g. This resulted in separation of debris and cells. Myelin debris was removed and the cells were washed using HBSS. Cells were then incubated for 1 min in Red Blood Cell lysis buffer (RBC lysis buffer; R7757; Sigma‐Aldrich) in order to remove contaminating red blood cells. Solution was then topped up by HBSS and centrifuged again. A2B5+ cells were removed using MACS. Following OPC (aged or young) MACS isolation, A2B5 negative cells were centrifuged (300 g, 5 min) and cell pellet was re‐suspended in DMEM‐F12 supplemented with 10% FBS (Fetal Bovine Serum) for ghost experiments, or OPC modified media for conditioned media experiments. Cells were plated densely in untreated suspension plates for overnight recovery. Next day, cells were detached from the suspension plates using ice cold HBSS supplemented with 5 mM EDTA. Cells were incubated in detachment solution in 4°C for 15–20 min and then removed using a pipette. The cell suspension was then centrifuged (300 g, 5 min) and re‐suspended in 90 μL MWB with 10 μL anti‐Rat‐CD11b magnetic micro beads (Miltenyi; cat#130‐105‐634) for every 10 million cells for 15 min in 4°C. Cells were washed using cold 8 mL MWB and then pelleted again (300 g, 5 min). Cells were then loaded to a MS column according to company instructions (Myltenyi).

Cells intended for ghost preparations were plated densely in 48 well plates, 40 K cells per well in OPCM (see table below) or DMEM‐F12 supplemented with 10% FBS. If serum based media was used, all comparisons were made to cells cultured in serum media as well. Cells were let to recover for 48 hr with/without TGFβ (Peprotech) and TGFβ inhibitor (SB431542, Tocris Bioscience)/BMP inhibitor (LDN‐193189, CELL guidance systems) before media was completely removed and ddH2O was added. Plate was immediately placed in dry ice for instant freeze. Plates were later kept for short term in −20°C, or long term in −80°C. Before use, plates were defrosted and the water was removed completely before new cells were plated on top of the ghosts.

For conditioned media experiments, cells were plated in density of 40,000 cells per well onto 24 well plates using OPC media (OPCM). After overnight recovery, media was completely removed and replaced with fresh OPCM supplemented with either TGFβ (Peprotech) or TGFβ inhibitor (SB‐431542, Tocris). Cells were cultured for 48 hr, afterwards media was removed, filtered using 22 μm filters to remove any cell debris and was frozen immediately using dry ice. Conditioned media was stored in −80°C until use.

### 2.4 Cells immunohistochemistry

At the end of each in vitro experiment, cells were fixed by adding 4% PFA to each well and incubating in RT for 10 min. Cells were then washed twice using PBS (5 min each wash, RT, shaking). Cell nuclei were labeled with 2 μg/mL Hoechst 33342 (Sigma). Before applying primary antibodies, cells were blocked using 5% normal donkey serum (NDS) for 20 min, RT. For intracellular staining, 0.1% Triton was added to the blocking solution. Primary antibodies (see Table 1) were diluted in PBS+ 5% NDS and samples were incubated overnight in 4°C. Following incubation, samples were washed twice with PBS (10 min, RT, shaking) and secondary antibodies were added. Secondary antibodies were diluted in PBS+ 5% PBS, and samples were incubated in RT for 2 hr. Following incubation, samples were washed 3 times with PBS, 10 min each time. Second wash included Hoechst 33342 for nuclear staining (2 ng/mL). Image acquisition was performed using a Leica‐SP5 microscope (Leica) and LAS software (Leica) or a Zeiss Observer A1 inverted microscope (Zeiss) and Zeiss Axiovision software. For each well (in 48 well plate) 3–4 images were taken in randomly selected areas. Further image processing and analysis was performed using the ImageJ software package. Images quantification was done using CellProfiler software (Broad Institute, Harvard University). In total, for each condition, a minimum of 100 cells were counted.

**Table 1:** Primary antibodies

Antibody	Dilution	Vendor	Catalog#
Mouse anti‐A2B5 (IgM)	1:300	Milipore	MAB312
Mouse anti‐CD11B	1:300	Serotec	MCA275R
Mouse anti‐CNPase	1:300	Abcam	ab6319
Mouse anti‐O4 (IgM)	1:1000	R&D systems	MAB1326
Rabbit anti‐NG2	1:300	Milipore	MAB5320
Goat anti‐IBA1	1:500	Abcam	ab5076
Goat anti‐OLIG2	1:500	R&D systems	AF2418
Chicken anti‐GFAP	1:1000	Abcam	ab4674

### 2.5 qRT‐PCR

RNA was isolated from acutely purified OPCs and microglia or from cultured OPCs and microglia using Qiagen RNeasy Micro Kit, or Directzol RNA MicroPrep Kit (Zymo Research; cat#R2061). Isolated RNA was immediately frozen using dry ice and was further stored in −80°C. RNA quantities were measured using Nanodrop 2000 (Thermo Scientific). cDNA was generated using Qiagen QuantiTect Reverse Transcription Kit according to the instructions of the manufacturer (Qiagen; 205310). For RT‐qPCR, primers were acquired from KiCqStart SYBR Primers (SIGMA‐ALDRICH) and used at a concentration of 300 μM (see Table 2). Primer efficiency was validated for all primers (90%–110%). cDNA, primers, and the Syber Green Master Mix (Qiagen; 204,141) were mixed as instructed by the manufacturer, and RT‐qPCR and melting curve analysis were performed on Life Technologies Quantstudio 6 Flex Real‐Time PCR System. Fold changes in gene expression were calculated using the delta delta Ct method in Microsoft Excel using TATA box binding protein (*Tbp*) as a control gene. Statistical significance was determined using two‐tailed unpaired *t* tests assuming equal variances.

**Table 2:** RT‐qPCR primers

Gene name	Sequence
*Cd44*	5′—AAGATTTTATCTCCAGCACC—3′ 5′—CTGTCTATATCAGTCATCCTGG—3′
*Cspg4*	5′—ACAAGCTCAAGAATTTCCAC—3′ 5′—TGAAGTTCCCTGTAGTGTAAG—3′
*Fn1*	5′—AAGCCAATAGCTGAGAAATG—3′ 5′—AAGTACAGTCCACCATCATC—3′
*Tbp*	5′—CATCATGAGAATAAGAGAGCC—3′ 5′—GGATTGTTCTTCACTTTGG—3′

### 2.6 Statistics

Statistical analysis was performed on GraphPad Prism 7 (GraphPad Software, Inc.). Immunohistochemical staining was identified using CellProfiler and CellProfiller Analyst (Carpenter et al., 2006) was used for machine learning cell identification and results were compared using suitable statistical tests. If >2 groups were present, a one‐way analysis of variance (ANOVA) test was performed, followed by appropriate post test in order to compare individual groups (Dunnett). For all statistical analysis, differences between groups were considered significant at *p* value < .05. Graphs were produced using GraphPad Prism or RStudio.

## 3 RESULTS

### 3.1 Effects of microglial ECM on OPCs

**Figure 1:**
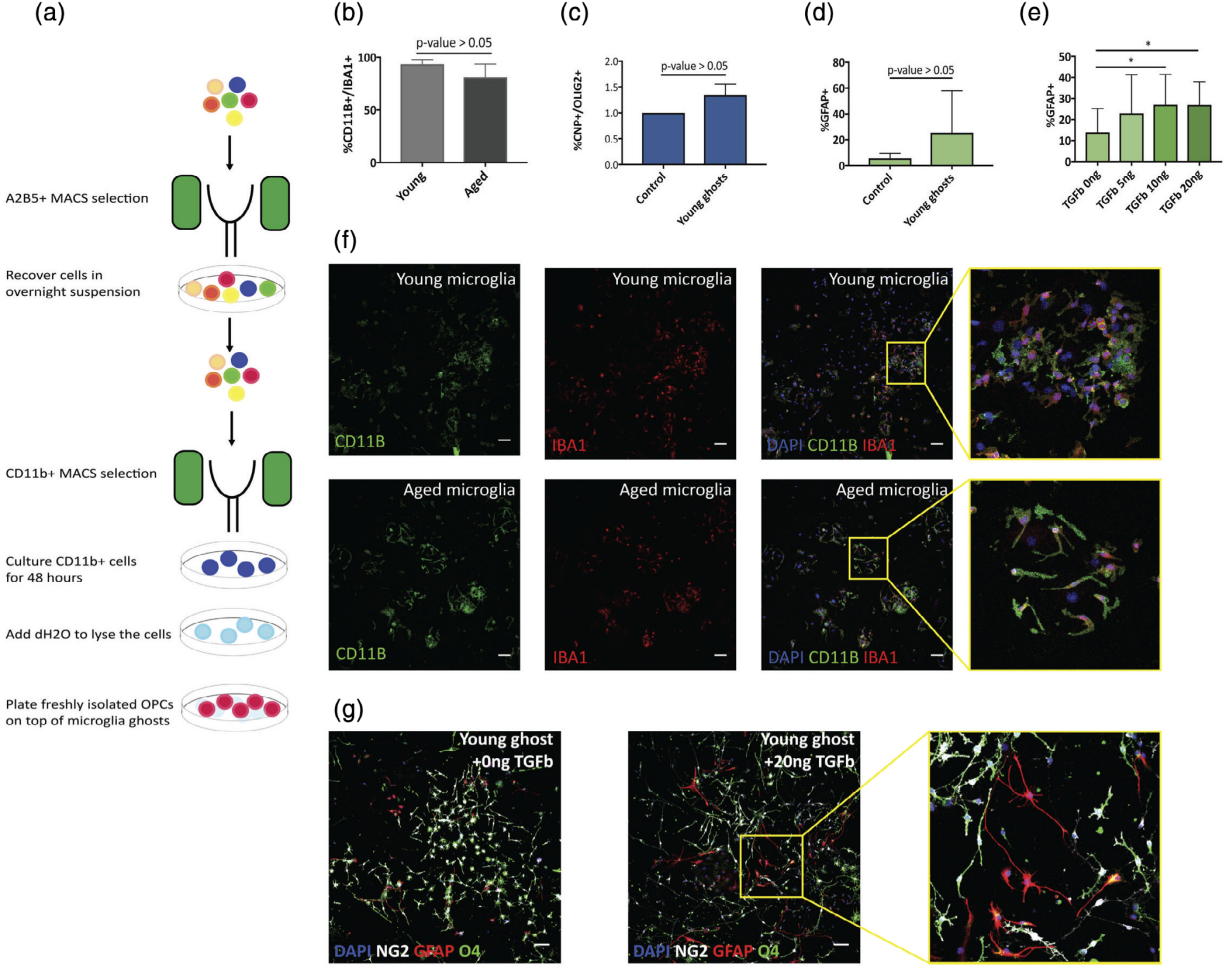
TGF β activated microglia surface molecules alter OPC differentiation in vitro. (a) A schematic description of the protocol used for generating microglia ghosts. A2B5 MACS was used to remove OPCs from cell suspension. After overnight recovery, microglia were isolated using CD11B labeled magnetic microbeads. Cells were plated, and lysed using ddH_2_O after 48 hr in culture. (b) 80% of the cells cultured from both young and aged adults following CD11B microbeads isolation were either CD11B+ or IBA1+ microglia. (c) Neonatal OPCs (isolated using A2B5 MACS) cultured on young microglia ghosts do not show any change in differentiation capacity (*n* = 3, *p* value > .05). (d) Neonatal OPCs cultured on top of neonatal microglia ghosts, show a not significant change in GFAP expression (ratio paired *t* test, *n* = 5, *p* value > .05), with specific culture presenting high percentages of astrocyte formation. (e) Neonatal OPCs cultured on top of TGFβ1 treated young microglia ghosts show significant increased expression of GFAP (one way ANPVA, *n* = 4, *p* value < .05) in a dose dependent manner. (f) Representative images of cultured microglia (young and aged). (g) Representative images of OPCs cultured on top of control PDL or aged microglia ghosts. OPCs were cultured for 4–5 days in serum free medium, and were stained for GFAP (red) to identify astrocytes formation and NG2 (white) and O4 (green) to identify OL lineage cells (scale bar represents 100 μm) [Color figure can be viewed at wileyonlinelibrary.com]

To investigate the potential effect of microglia ECM molecules on OPC differentiation in isolation of possible effects mediated by secreted factors, we developed a protocol that yielded preparations of microglia membrane molecules and ECM depositions on which OPCs could be cultured. We termed these preparations microglial “ghosts” (Figure 1a). In brief, whole brains of young adult and aged rats were lysed and single cell suspensions generated. First, MACS (Magnetic Activated Cell Sorting) for A2B5+ cells was used to remove OPCs. Microglia were then isolated using MACS for cells expressing the microglial marker CD11B+. We established primary microglia cultures (>80% CD11B+ or IBA1+ cells) from both neonatal/young rats, as well as aged rats (Figure 1b,f). For ghost preparations, microglia were cultured in PDL coated plates for 48 hr before being lysed by ddH_2_O (see Figure 1a for schematic description). This yielded substrates comprising microglia membrane bound molecules and microglia‐associated ECM, but lacking active microglia (the method is similar to that used for astrocytes by Keough et al., [2016]). Neonatal OPCs were then plated on the microglial “ghosts” generated from microglia. When OPCs were plated on ghosts from naïve young microglia there was no significant change in OL or astrocyte generation as shown by CNP (Figure 1c) and GFAP (Figure 1d) staining when compared with PDL control wells. We next asked whether ghosts generated from activated microglia would affect OPCs properties. We treated microglia with TGFβ since (a) it is known to alter ECM production in other cell types (Ignotz & Massagué, 1986; Laping et al., 2002), (b) microglia express high levels of TGFβ receptors (Lavin et al., 2014), and (c) it is present in MS lesions (De Groot, Montagne, Barten, Sminia, & Van Der Valk, 1999). We treated microglia with increasing concentrations of TGFβ (0–20 ng/mL) for 48 hr prior to generating ghosts. OPCs plated on ghosts derived from microglia treated with 10–20 ng/mL of TGFβ showed significantly increased expression of GFAP and astrocyte formation when compared with PDL coated control wells (*p* value < .05, *n* = 5) (Figure 1e,g). Although the origin of the astrocytes in the culture cannot be unequivocally stated to arise from OPCs and not from a minority of contaminating astrocytes, it is likely to be the former since (a) isolation of OPCs using A2B5 antibodies does not result in significant astrocyte contamination and (b) all of the astrocytes had a morphology (long separated processes) typical of astrocytes generated by OPCs (Raff, Miller, & Noble, 1983; Tanner, Cherry, & Mayer‐Proschel, 2011).

### 3.2 TGFβ alters neonatal microglial‐associated ECM

**Figure 2:**
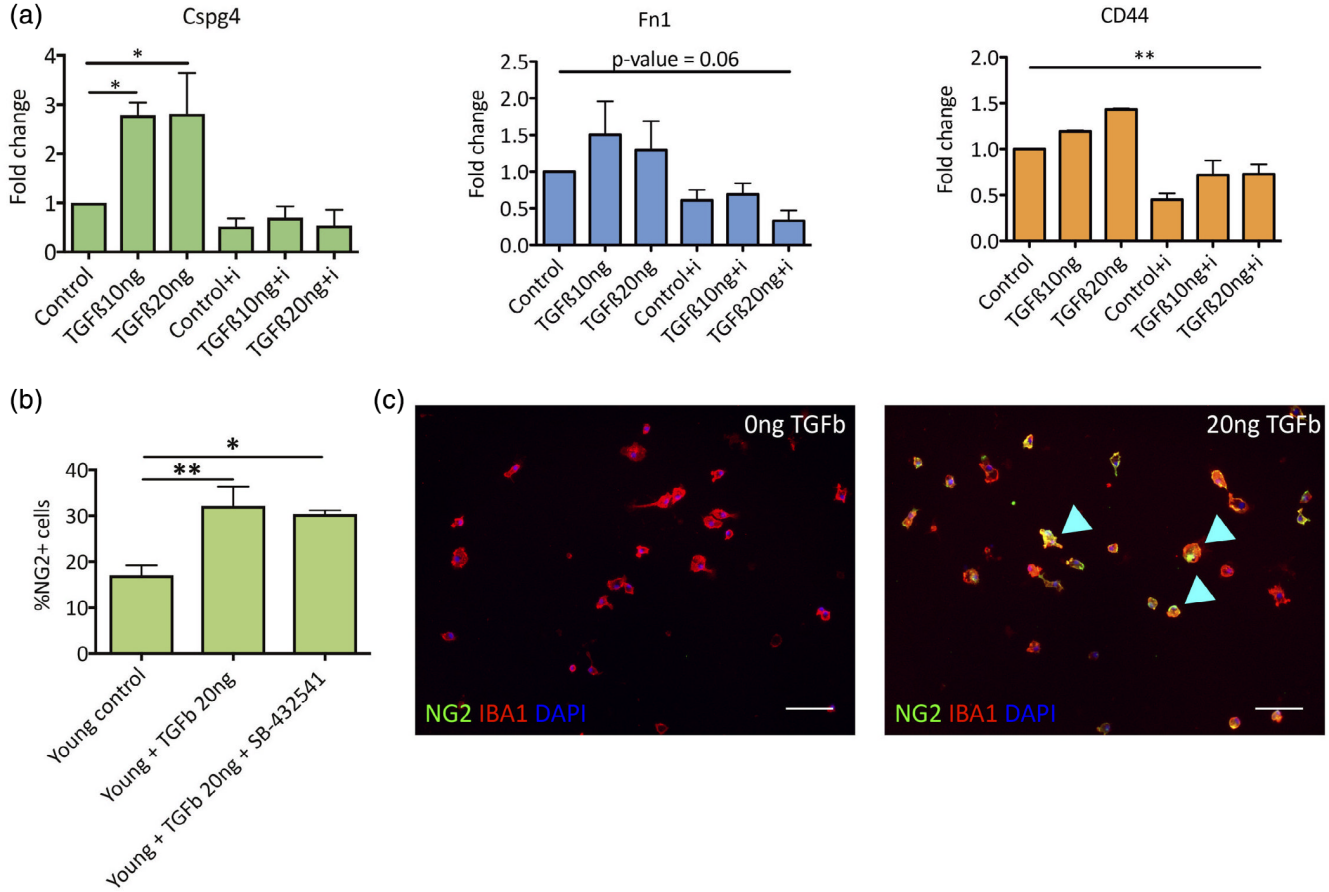
TGFβ alters the expression of surface molecules in young microglia. (a) Young microglia treated in vitro (48 hr) with TGFβ1 exhibit increase in mRNA expression of multiple surface antigens, such as *Fn1*, *Cspg4*, and *Cd44*, quantified by qRT‐PCR (*n* = 3). The increase in mRNA expression is eliminated in the presence of the TGFβ small molecule inhibitor (SB‐432541, 5 μM). (b) in vitro quantification of young microglia cultured in the presence of TGFβ1 (20 ng/mL) with or without TGFβ inhibitor (SB‐432541, 5 μM) (*n* = 4, *p* value < .01). (**c**) IHC of microglia treated with TGFβ co‐expressing NG2 (green) and microglia marker IBA1 (red) [Color figure can be viewed at wileyonlinelibrary.com]

We next asked if we could identify the changes elicited in microglia by exposure to TGFβ. The effect of TGFβ was tested on young microglia in vitro by measuring mRNA levels of *Cspg4*, *Fn1,* and *Cd44* (genes coding for the ECM molecules, NG2, Fibronectin, and CD44, respectively). qRT‐PCR revealed that 48 hr treatment with 10–20 ng/mL TGFβ1 induced an increase in the expression of these three genes (Figure 2a), an effect that was eliminated by the presence of TGFβ inhibitor SB‐431542 (5 μM) (Inman et al., 2002) (Figure 2a). We did not observe changes in cell viability when adding TGFβ inhibitor SB‐431542 (data not shown). These results are consistent with a previous study (Sugimoto et al., 2014), which showed that following stroke, rat microglia express NG2 on exposure to TGFβ.

We further verified this effect by immunostaining TGFβ1‐treated young microglia for NG2 (Figure 2b). As predicted, young microglia treated with TGFβ1 (20 ng/mL) for 48 hr in vitro showed a significant increase in the percentage of NG2+ cells (Figure 2b,c). The addition of TGFβ inhibitor SB‐431542 to culture did not reduce the number of NG2+ cells, in contrast to the reduction in mRNA levels (possibly due to longer half life of the protein versus the mRNA). We therefore concluded that TGFβ induced changes in the microglia‐generated ECM in a way which promoted astrocyte formation by OPCs.

### 3.3 Aged microglia show ECM signature similar to TGFβ treated microglia

**Figure 3:**
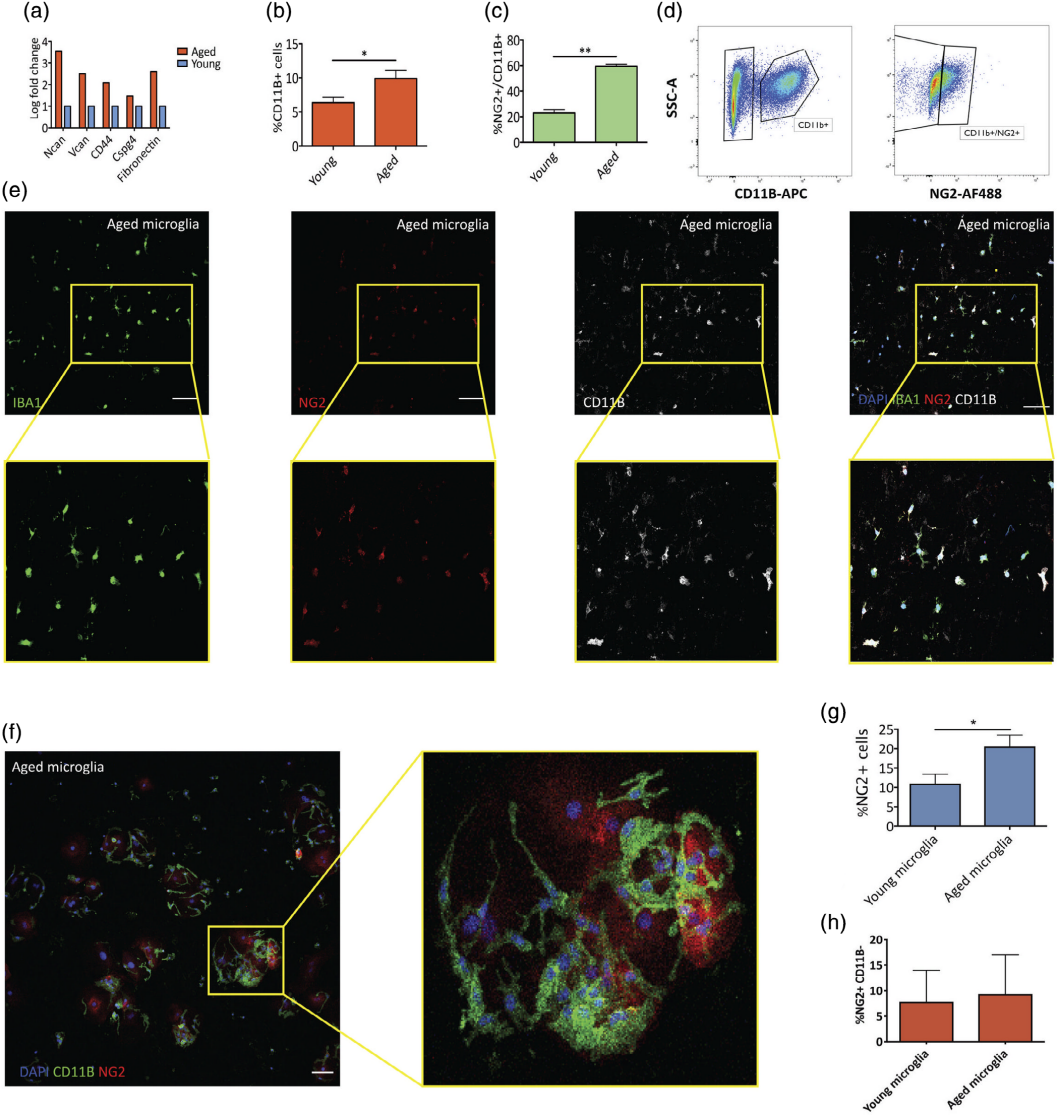
Changes in aged microglia surface molecules. (a) Published RNA‐seq data show increases in expression of multiple proteoglycan genes in aged microglia when compared with young microglia cells (Hickman et al., 2013). (b) FACS isolation of young (2–4 months old) and aged (18–22 months old) microglia using CD11B antibodies reveals a significant increase in microglia proportions in aged rats (paired *t* test, *p* value < .05, *n* = 3). (c) FACS analysis of the proportion of NG2+ cells out of total CD11B+ cells show a significant increase in aged rats (paired *t* test, *p* value <0.005, *n* = 3). (d) Example of FACS scatterplot of aged microglia. (**e**) Representative images of young and aged microglia cultured in vitro and stained for IBA1, CD11B (microglia markers), and NG2. (f) Small numbers of NG2+/CD11B− cells with a flat morphology were also detected. (g) Proportions of NG2+ are higher in aged microglia acutely isolated using CD11B MACS and cultured 24–48 hr in vitro (paired *t* test, *p* value < .05, *n* = 3). (h) There was no significant difference in the percentage of these cells between aged and young microglia preparations (scale bar represents 100 μm) [Color figure can be viewed at wileyonlinelibrary.com]

Since circulating TGFβ levels increase with ageing (Buckwalter et al., 2006; Carlson et al., 2009), we next tested whether aged microglia present similar ECM composition to TGFβ treated young microglia due to their long exposure to TGFβ throughout adult life. When exploring published RNA sequencing databases (Hickman et al., 2013), we identified multiple membrane associated molecules that show increased levels of expression in aged microglia (Figure 3a). Further analysis using Fluorescence Activated Flow Cytometer (FACS) revealed that aged rats have significantly higher numbers of microglia (labeled by CD11B) (Figure 3b), and a significantly higher percentage of NG2 positive labeled microglia (%CD11B+NG2+/CD11B+) (Figure 3c,d). We further verified this by culturing young and aged microglia following CD11B+ MACS sorting and confirmed that a significantly higher proportion of NG2+CD11B+ cells when compared with young microglia (Figure 3g). Even though NG2 is usually used for identifying OPCs in the CNS, our cells were not positive for OLIG2, a marker of oligodendrocyte lineage cells, nor did they show the morphology of oligodendrocyte lineage cells. In contrast, many of the NG2+ cells had microglial morphology, although some did not show high expression of CD11B (Figure 3e,f). Although there was no significant difference in the percentages of NG2+/CD11B− cells between cultures (Figure 3h), we cannot rule out the possibility of infiltration of other cell types, such as pericytes, which can express also NG2 (Özen et al., 2014).

### 3.4 Aged microglial ghosts recapitulate TGFβ treated microglia effects

**Figure 4:**
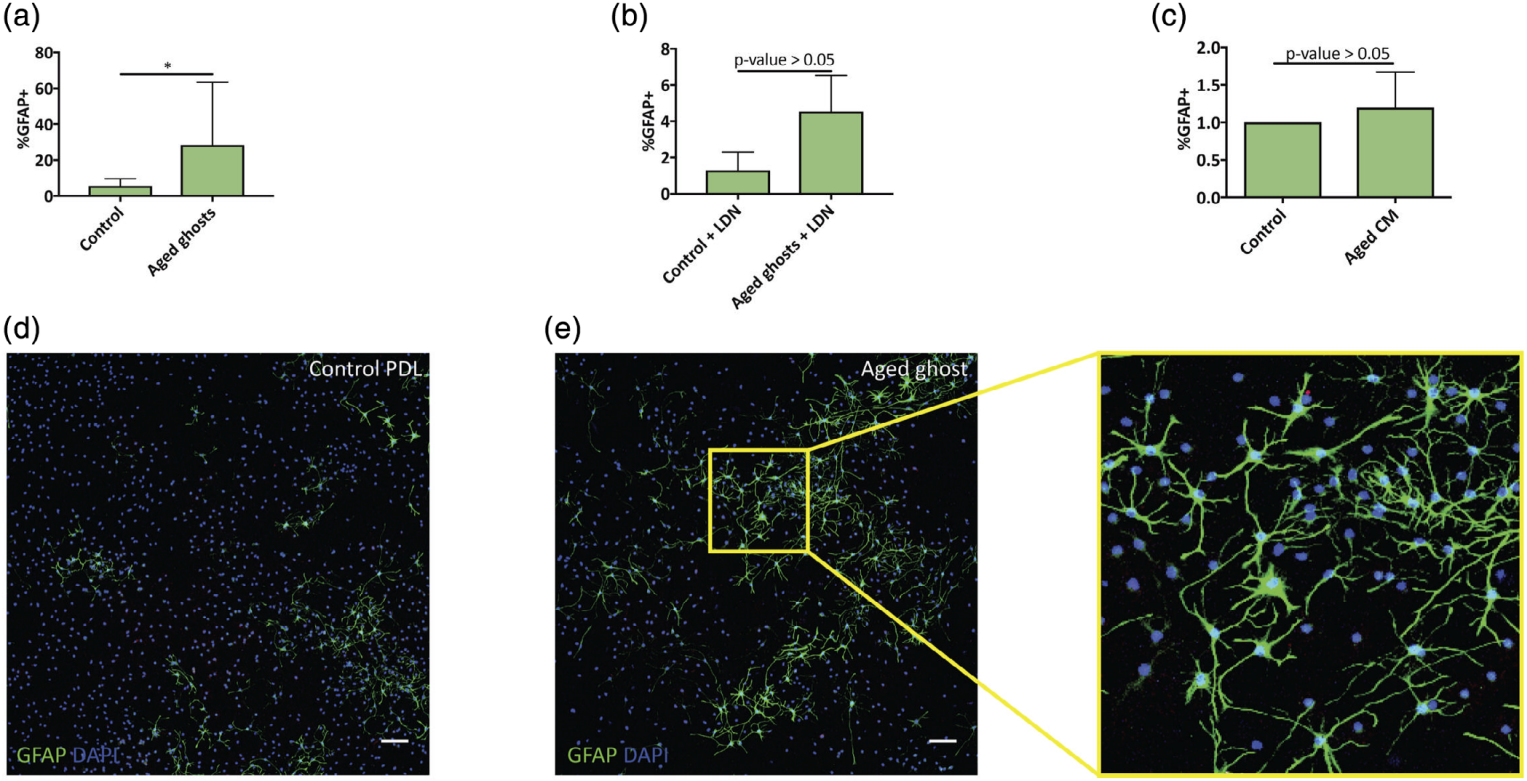
Aged microglia surface molecules alter OPC differentiation in vitro. (a) Neonatal OPCs cultured on top of aged microglia ghosts show significant increased expression of GFAP (ratio paired *t* test, *n* = 5, *p* value < .05). (b) Culturing neonatal OPCs on young or aged microglia ghosts in the presence of BMP inhibitor (LDN‐193189) blocks the differentiation into astrocytes and GFAP expression (ratio paired *t* test, *n* = 3, *p* value < .05). (c) OPCs cultured with aged microglia conditioned media (CM) do not show upregulation in astrocyte formation (*p* value > .05, *n* = 3). (d,e) Representative images of OPCs cultured on top of control PDL or aged microglia ghosts. OPCs were cultured for 4–5 days in serum free medium, and were stained for GFAP (green) to identify astrocytes formation (scale bar represents 100 μm) [Color figure can be viewed at wileyonlinelibrary.com]

Having found increased expression of NG2 and other ECM molecules in aged microglia (Figure 3), we asked whether this was enough to elicit changes in OPC differentiation in a similar fashion to the effect we observed when using TGFβ treated young microglia. Similar to the previous experiment, we cultured aged microglia for 48 hr prior to microglial ghost generation. OPCs were then plated on top of these ghosts and the extent of astrocyte formation was assessed by staining for GFAP. OPCs plated on top of aged microglia ghosts had significantly increased generation of astrocytes when compared with PDL control wells (Figure 4a,d,e). We then asked whether this effect could be recapitulated using aged microglia conditioned media. For this, microglia were cultured for 48 hr before retrieving the conditioned media. This conditioned media was mixed in a 1:1 ratio with fresh media. OPCs that were grown in medium conditioned by aged microglia did not show an increase in astrocyte formation (Figure 4c), supporting the inference that ECM components produced by microglia were responsible for the differentiation effects described rather than secreted factors. The lack of effect using conditioned media could also be due to technical reasons such as shorter conditioning time leading to lower concentrations of secreted molecules in the conditioned media.

As BMP signaling has previously been shown to promote the generation of astrocytes by OPCs (Mabie et al., 1997), we tested whether this might be the mechanism underlying the astrocyte formation by ghosts derived from aged microglia. OPCs cultured on aged ghosts in the presence of LDN‐193189, a small molecule BMP inhibitor, did not show increased astrocyte formation (Figure 4b), suggesting that the effect exerted by the microglia ghosts was via the activation of BMP signaling in OPCs.

### 3.5 Aged microglia express NG2 following demyelination

**Figure 5:**
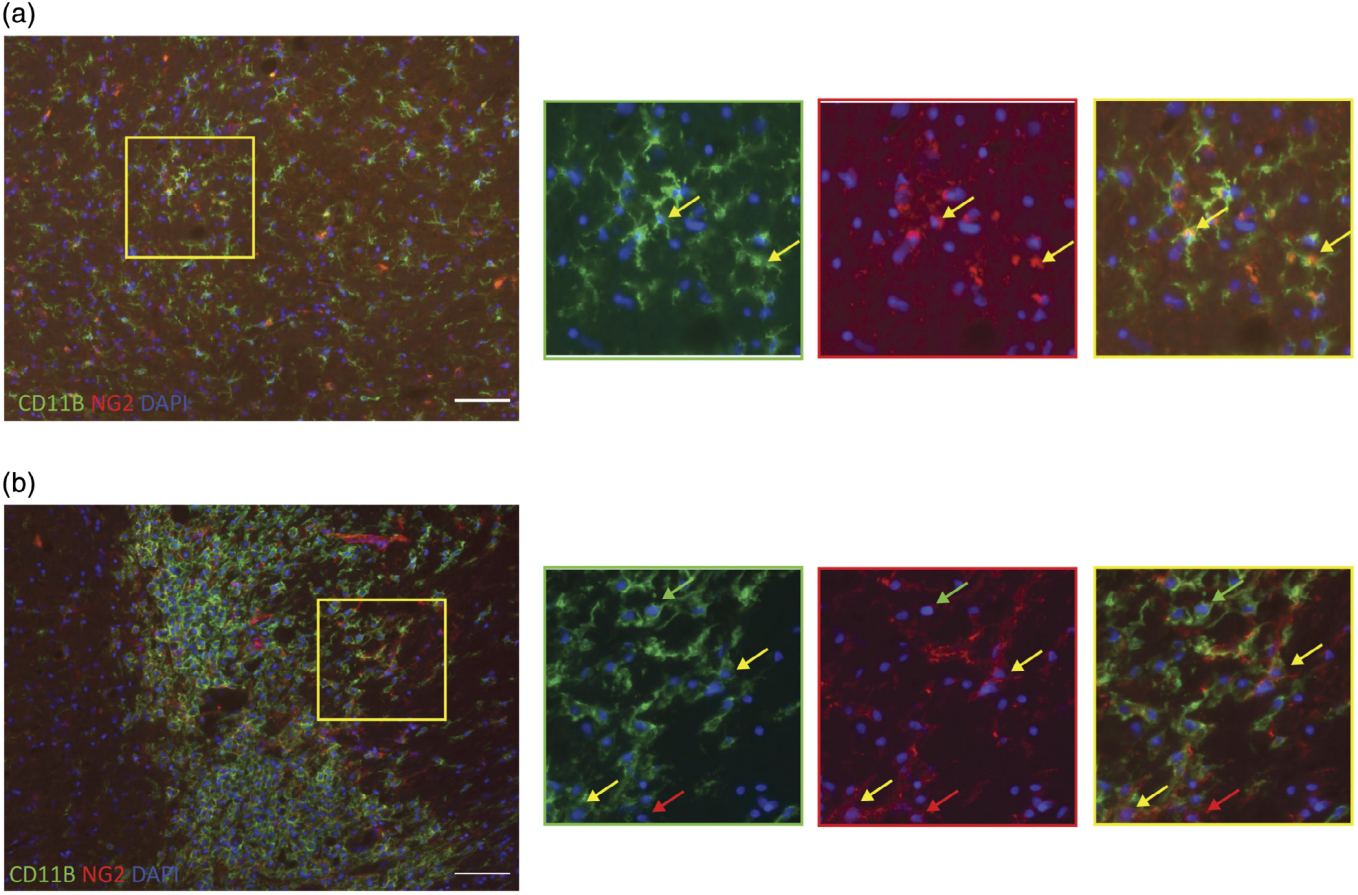
NG2 positive microglia present in NAWM and demyelinating lesions in aged rats. (a) NAWM (normal appearing white matter) in aged rats was imaged for CD11B (identification of microglia) and NG2. Several microglia were double labeled. (b) 5 days after the induction of demyelinating lesion, multiple microglia cells expressed high reactivity to NG2 staining. Green arrows represent CD11B + NG2− cells, red arrows point out CD11B − NG2+ cells and yellow arrows point to CD11B + NG2+ cells [Color figure can be viewed at wileyonlinelibrary.com]

We next tested the expression of NG2 by aged microglia in vivo. Focal areas of spinal cord white matter demyelination was induced in aged rats (>18 months) by the injection of ethidium bromide (EB). At 5 days post lesion‐induction many cells were positive for both CD11B and NG2 within the lesion area (Figure 5b). There were also a small number of double positive cells in normal appearing white matter of aged rats (Figure 5a).

### 3.6 TGFβ directly inhibits OPC differentiation

**Figure 6:**
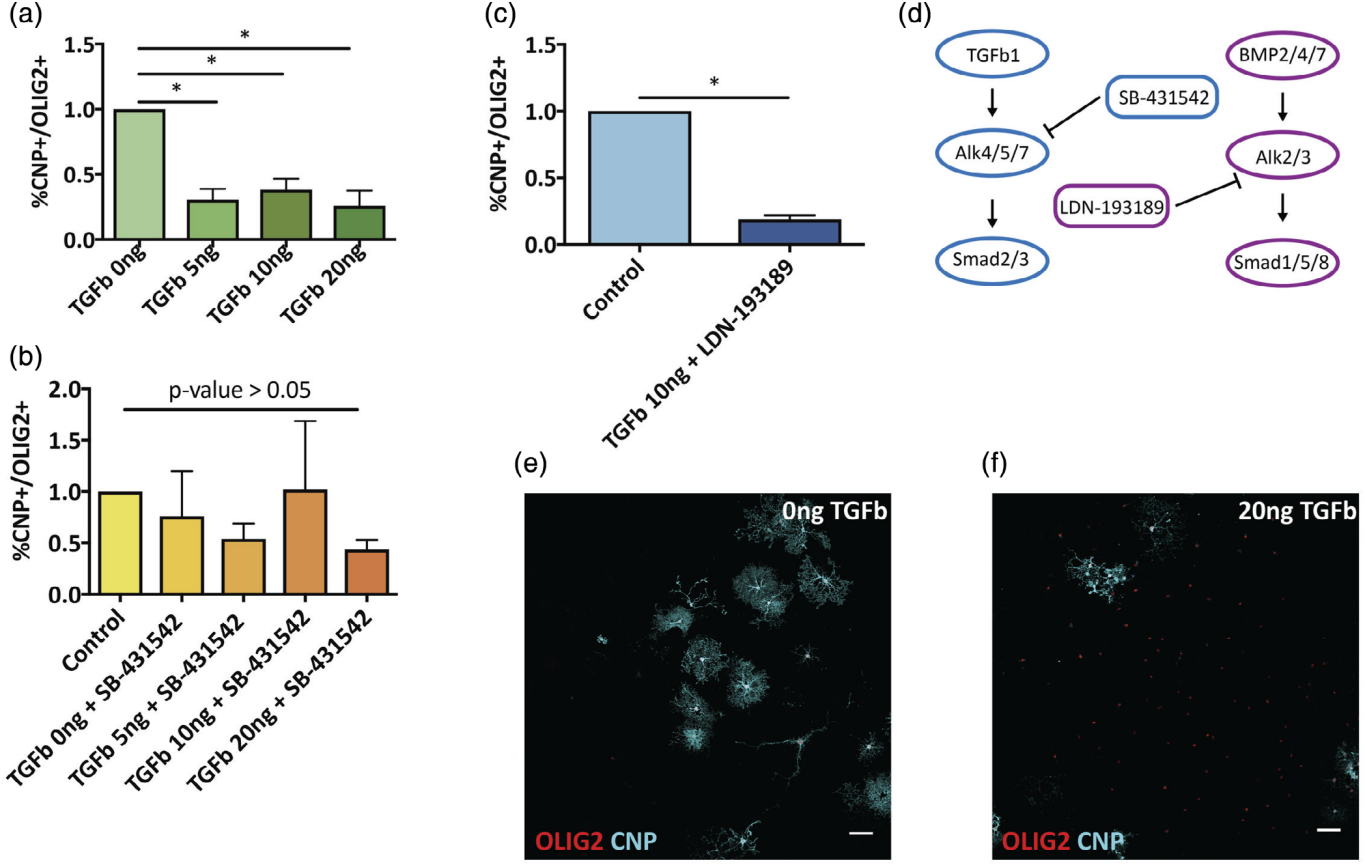
TGFβ directly inhibits OPC differentiation in vitro. (a) OPCs were cultured with various concentrations of TGFβ (0–20 ng/mL) for 6 days in vitro. Differentiation was assessed by CNP staining. The presence of TGFβ in culture media resulted in significant reduction in OPC percentage of CNP+/OLIG2+ cells (out of total OLIG2+ cells, normalized to control) (one‐way ANOVA, *n* = 3, *p* value < .05). (b) When cells were cultured with TGFβ and the addition of TGFβ small molecule inhibitor (SB‐431542; 5 μM) differentiation inhibition was eliminated (one‐way ANOVA, *n* = 3, *p* value > .05). (c) OPCs cultured with 10 ng/mL TGFβ and the presence of BMP pathway inhibitor (LDN‐193189; 5 μM) showed again significant decrease in differentiation capacities (paired *t* test, *n* = 2, *p* value < .05). (d) Schematic description of pathways blocked by SB‐431542 and LDN‐193189. (e) Representative images of OPCs in vitro stained for OLIG2 (red) and CNP (cyan) with or without the addition of TGFβ [Color figure can be viewed at wileyonlinelibrary.com]

The effects of TGFβ on microglia surface antigens prompted us to test the direct effects of TGFβ on OPC differentiation. Previous studies have identified TGFβ as a promoter of OPC differentiation (McKinnon, Piras, Ida, & Dubois‐Dalcq, 1993; Palazuelos, Klingener, & Aguirre, 2014). To test this, neonatal OPCs (isolated using A2B5 MACS) were cultured in serum free media, which was supplemented by TGFβ1 (5–20 ng/mL), with or without the TGFβ inhibitor SB‐431542 (5 μM) (Figure 6). Cells were cultured for 6 days in vitro before differentiation was assessed by immunostaining with the oligodendrocyte differentiation marker CNPase. In the presence of 20 ng/mL TGFβ1, there was a significant reduction in OPC differentiation compared with controls (Figure 6a), an effect that was eliminated by the TGFβ inhibitor (SB‐431542) (Figure 6b). We further tested the specificity of TGFβ1 effects on OPCs, by adding the BMP inhibitor LDN‐193189 to cultures treated with TGFβ1 (Figure 6c). LDN‐193189 did not ameliorate the effects of TGFβ. Since the two inhibitors act through different receptors, this enabled us to identify the pathway by which TGFβ1 affected OPCs (Figure 6d).

## 4 DISCUSSION

OPCs can differentiate into oligodendrocytes, Schwann cells, and, albeit infrequently, into astrocytes (Assinck et al., 2017; Zawadzka et al., 2010). Here, we show that ECM deposited by TGFβ treated and aged microglia can directly alter the fate choice of OPCs, directing them toward an astrocytic fate. The effect of ECM and cell to cell contact on OPCs has been shown in relation to other cell types. For example, neurons can control their own myelination, both by secreting neurotransmitters (Gautier et al., 2015), but also by expressing specific surface molecules that inhibit OLs from wrapping them with myelin (Redmond et al., 2016). Similarly, experiments with astrocytes show that they generate ECM that prevents OPC differentiation in vitro molecules (Keough et al., 2016). Here, we show that microglial ECM can influence cell fate decisions by OPCs, and that this, in part, depends on BMP signaling. BMP signaling relies on the phosphorylation of SMAD1/5/8 by ALK2/3 (Figure 5d). Using small molecule inhibitor (LDN‐193189) which specifically targets ALK2/3, we were able to block the induction of GFAP in OPCs cultured on microglia ghosts. These results are consistent with previous studies which have shown that BMP signaling induces astrocytic fate in OPCs and other CNS progenitor cells (Gross et al., 1996; Mabie et al., 1997; Petersen et al., 2017).

Using microglia ghost preparations, we have ruled out the possible effects of microglia secreted factors, as there are no viable cells and secreted factors are removed by aspirating the cell media and replacing it with ddH_2_O. The use of conditioned media from aged microglia did not affect the behavior of the OPCs, in contrast to previous reports using conditioned media from activated microglia (Miron et al., 2013; Yuen et al., 2013). This could be due to limited time the microglia were cultured before media was taken, as it is possible that this was not enough time for substantial concentration of factors to be released into the media in sufficient concentrations. Moreover, in previous reports the microglia were activated by potent agents (e.g., LPS) which are likely to cause significantly higher expression of secreted factors. In contrast, treatment with TGFβ, an anti‐inflammatory cytokine, that we have used in this study promotes production of ECM and membrane bound molecules (Laping et al., 2002).

### 4.1 TGFβ changes the microglial micro‐niche

Since naïve young microglia ghosts did not significantly affect OPC differentiation (Figure 1b,c), unlike the effects shown previously by naïve astrocytes (Keough et al., 2016), we considered whether activated microglia more similar to a state likely to be present in demyelinated lesions would have a different effect. We used TGFβ to activate the microglia since it has been shown previously to be present in elevated levels in MS lesions (De Groot et al., 1999) and circulating blood of MS patients (Nicoletti et al., 1998). Moreover, TGFβ is known to play a pivotal role in microglia development, and microglia express higher levels of the TGFβ receptors than other tissue resident macrophages (Butovsky et al., 2014; Hickman et al., 2013; Lavin et al., 2014). TGFβ has been shown to promote the expression of fibronectin, which inhibits remyelination (Stoffels et al., 2013) and other ECM molecules in different cell types (Ignotz & Massagué, 1986; Laping et al., 2002; Roberts et al., 1988). Following stroke, TGFβ is linked to upregulated NG2 expression in microglia (Sugimoto et al., 2014). Expression of NG2 in microglia has been associated previously with lower phagocytic capacities (Zhu et al., 2012). Moreover, microglia in NG2 knockout mice show increased expression of neurotrophic factors and decreased expression of pro‐inflammatory markers following facial nerve axotomy (Zhu et al., 2016).

These findings, in combination with rising levels of TGFβ with ageing (Carlson et al., 2009), position TGFβ as a leading candidate for explaining the underlying mechanisms that alter microglia membrane bound proteins. Consistent with these studies, we also show that TGFβ can induce the expression of NG2 in microglia in vitro, and furthermore can induce an “aged like” membrane bound proteins signature. TGFβ exerts its effects in cells through phosphorylation of SMAD2/3 by ALK4/5/7. Using ALK4/5/7 specific small molecule inhibitor (SB‐431542) (Inman et al., 2002; Laping et al., 2002), we show that these effects are abolished and thus the increased expression of NG2 by microglia is prevented. We therefore conclude that TGFβ alters microglia in such a way that it activates BMP signaling in OPCs, driving them toward astrocyte generation. Although our primary focus has been on NG2 expression, we do not exclude the involvement of other ECM molecules in the effects described. RNA sequencing databases of aged and young microglia show increase in multiple ECM molecules in aged microglia, including fibronectin and VCAM (Hickman et al., 2013). Our identification of NG2 expression in microglia is supported by previous reports that which show that NG2 expression plays a role in microglia activation in pathological conditions (Sugimoto et al., 2014; Zhu et al., 2016), even though NG2 is generally regarded as reliable marker for OPCs (Nishiyama, Chang, & Trapp, 1999). Our data and those of others thus confirm that NG2 does not exclusively identify OPCs.

### 4.2 TGFβ directly inhibits OPC differentiation

Since TGFβ was shown to alter OPC differentiation via its effects on microglia, we further tested whether TGFβ could affect OPCs directly. Previous reports claimed that TGFβ promotes OPC differentiation (McKinnon et al., 1993; Palazuelos et al., 2014). In contrast, we find evidence for direct inhibition of OPC differentiation by TGFβ in vitro. There are a number of possible explanations. The article by McKinnon et al. (1993) showed an inhibition of proliferation which likely accounted for an indirect increase in differentiation. In the article by Palazuelos et al. (2014), the TGFβ effects reported were on cells at a more mature stage of development (already expressing CNP) than the cells used in our study. Thus, it is possible that TGFβ has different effects in different stages of OPC differentiation. This is supported by previous studies in which higher levels of TGFβ were associated with lower levels of MBP following focal demyelination (Kotter, Zhao, Van Rooijen, & Franklin, 2005).

Our findings reveal TGFβ as a potential therapeutic target for enhancement of remyelination in aged patients. These results support previous studies implicating the harmful effects of TGFβ in ageing, and specifically in regeneration (Buckwalter et al., 2006). Future experiments targeting TGFβ by, for example, the administration of TGFβ pathway inhibitors such as the FDA approved compound Losartan, could be undertaken to assess their effects on remyelination in aged animals (Habashi et al., 2011; Holm et al., 2011).

In summary, we report a novel role for microglial ECM in determining OPC differentiation fate. As microglia constitutes the majority of the cells in most demyelination lesions (especially during the early stages of remyelination), these findings could prove important in the design of remyelination therapies. Moreover, we identify TGFβ as a key cytokine which can exert inhibitory effects on the differentiation of OPCs, an effect that increases with ageing.
